# MOSAIC: an online database dedicated to the comparative genomics of bacterial strains at the intra-species level

**DOI:** 10.1186/1471-2105-9-498

**Published:** 2008-11-27

**Authors:** Hélène Chiapello, Annie Gendrault, Christophe Caron, Jérome Blum, Marie-Agnès Petit, Meriem El Karoui

**Affiliations:** 1 INRA UR1077, Unité Mathématique, Informatique & Génome, Domaine de Vilvert, 78352, Jouy-en-Josas, France; 2 INRA UR888, Unité des Bactéries Lactiques et Pathogènes Opportunistes, Domaine de Vilvert, 78352, Jouy-en-Josas, France

## Abstract

**Background:**

The recent availability of complete sequences for numerous closely related bacterial genomes opens up new challenges in comparative genomics. Several methods have been developed to align complete genomes at the nucleotide level but their use and the biological interpretation of results are not straightforward. It is therefore necessary to develop new resources to access, analyze, and visualize genome comparisons.

**Description:**

Here we present recent developments on MOSAIC, a generalist comparative bacterial genome database. This database provides the bacteriologist community with easy access to comparisons of complete bacterial genomes at the intra-species level. The strategy we developed for comparison allows us to define two types of regions in bacterial genomes: backbone segments (i.e., regions conserved in all compared strains) and variable segments (i.e., regions that are either specific to or variable in one of the aligned genomes). Definition of these segments at the nucleotide level allows precise comparative and evolutionary analyses of both coding and non-coding regions of bacterial genomes. Such work is easily performed using the MOSAIC Web interface, which allows browsing and graphical visualization of genome comparisons.

**Conclusion:**

The MOSAIC database now includes 493 pairwise comparisons and 35 multiple maximal comparisons representing 78 bacterial species. Genome conserved regions (backbones) and variable segments are presented in various formats for further analysis. A graphical interface allows visualization of aligned genomes and functional annotations. The MOSAIC database is available online at .

## Background

The increasing number of publicly available, completely sequenced bacterial genomes provides an opportunity for original comparative genomics approaches, especially at short-term evolutionary scales. During the last decade, several algorithms have been developed to respond to the challenging task of aligning whole genomes at the nucleotide level (see, for instance, references [1-3], and [4]). Some algorithms, such as MGA, are limited to collinear genomes [1]. Others, however, such as the MAUVE aligner [2], allow alignments of multiply rearranged (i.e., inverted or translocated) genomic sequences. These powerful tools enable novel exploration of bacterial genome structure and evolution. However, the use of these algorithms presents certain difficulties in practice. First, adjustment of alignment parameters is not straightforward. Second, no statistical or empirical criteria are available to evaluate the quality of genome alignments. Third, displaying, browsing, and analyzing genomic sequence alignments are challenging (for review see reference [5]).

To provide easy access to the genomic structure of closely related bacterial species, we have developed a comprehensive database termed MOSAIC. Several resources have been made available in the area of bacterial comparative genomics (for a review see [6]), but most are dedicated to a given species or group of species (e.g., the Enterix tools [7]). Moreover, these resources are often restricted to pairwise genome comparisons (e.g., xBASE2 [8]). The MOSAIC database is a generalist resource that aims to provide easy access to pairwise and multiple bacterial genome comparisons at the intra-species level. Compared to the previous release [9], the new version of MOSAIC includes several improvements in comparison strategies and database content that allow for a broader definition of the nature of conserved and variable segments in bacterial genomes. Genomes are now extracted from the EBI Genome Reviews database [10] instead of the NCBI RefSeq database [11]. Comparisons are based on two genome aligners: MGA [1] for collinear genomes and MAUVE [2] for rearranged genomes. To facilitate interpretation, genome alignments are post-processed to define backbone segments (i.e., regions conserved in all compared strains) and variable segments (i.e., regions that are either specific to or variable in one of the aligned genomes). These segments are easily accessible through the MOSAIC interface, which allows browsing and genome comparison visualization using three graphical modes: Genome Comparison Viewer, Physical Linear Map, and Circular Map.

## Construction and content

### Comparison strategy

**Figure 1:**
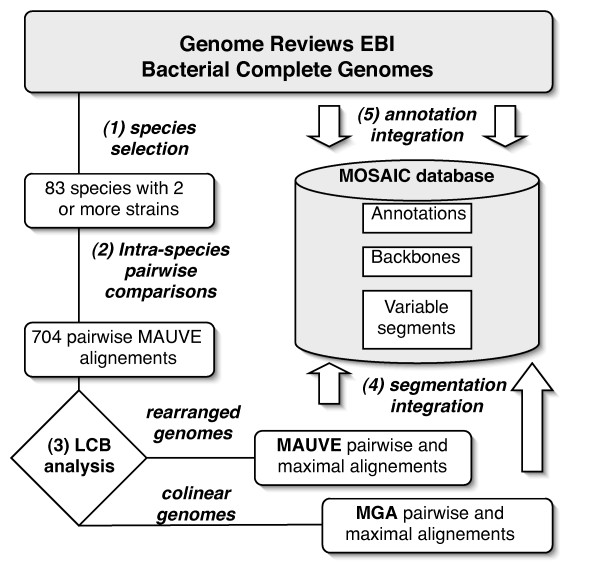
**Comparison strategy used to construct the MOSAIC database**. When at least two strains of a species are sequenced, genomes are first extracted from the GR database [step (1)]. Systematic intra-species pairwise genome alignments are then performed with MAUVE [step (2)]. A test for the presence of rearrangements in the pair of aligned genomes is then applied using the number and size of defined MAUVE LCBs (Locally Collinear Blocks) in step (3). The LCB analysis permits genomes to be designated either as collinear or rearranged. The collinear genomes are realigned with MGA (at least pairwise, and possibly with maximal multiple alignment if sequences of more than two strains are available). Rearranged genomes are aligned with MAUVE (maximal multiple alignments). Finally, MGA and MAUVE alignments are post-processed and genomes are segmented into backbone regions and variable segments in step (4), and integrated in the MOSAIC database together with annotations in step (5).

The comparison strategy is summarized in Figure 1.

#### Extraction from the Genome Reviews database [10]

Bacterial genomes are extracted from the Genome Reviews (GR) database [10]. We chose this resource because it provides standardized, enriched, and up-to-date annotations while maintaining cross-references to primary submissions. Upgraded annotations are derived from the integration of data from many sources, including the EMBL Nucleotide Sequence Database, the UniProt Knowledgebase, the InterPro Protein Domain and Families database, the Gene Ontology Annotation Database, and others. All bacterial species for which complete genome sequences of at least two strains (according to species nomenclature) are available were chosen. This process resulted in the selection of 83 species from GR database release 96 (09/23/2008), corresponding to 331 genomes. Plasmids and extra-chromosomal sequences were removed at this point.

#### Systematic intra-species pairwise comparisons

The comparisons were performed using the MAUVE genome aligner version 1.2.3 [2]. The reference genome was arbitrarily chosen to be the shortest. MAUVE parameters were chosen as follows. As a first step, parameters were tested on a pairwise comparison of collinear genomes to choose parameter values. The MGA alignment of *Escherichia coli *strains MG1655 and O157:H7 Sakai was chosen as a reference, as this had been constructed using validated parameters in the previous release of the MOSAIC database [9]. Three parameters were adjusted for performing a MAUVE alignment. First, the "minimal recursive gap length" was increased from the default value (200 nt) to 5000 nt, as this reduced the number of small adventitious backbone segments (see Table 1a). Second, the "locally collinear block (LCB) weight" was increased from the default value (57 nt) to 5000 nt, as this reduced the number of adventitious LCBs from 113 to 4 (see Table 1b). The residual fragmentation of these two collinear genomes into four LCBs arose because of the presence of a 6.7 kb translocation, corresponding to a set of genes common to two bacteriophages (DLP12 in MG1655 and Sp8 in O157:H7 Sakai). Finally, the default seed size (15) was increased to 19, as this permitted a closer correspondence to the seed size used in MGA. Indeed, MGA alignments stored in the MOSAIC database are performed using a seed of 50 in the first step, and a seed of 20 in the second step. MAUVE alignments generated with min_rec_gap_len = 5000 and weight = 5000, and seed values of 15 or 19, were compared to MGA alignments in two ways. First, the global number of kilobase pairs (kb) in the "false backbone" (i.e., belonging to the variable segments of MGA but found in the backbone of the MAUVE alignment) was counted. This was 30.4 kb for seed 15 and 24.8 kb for seed 19. Second, the reciprocal global number of kb in "false variable segments" (i.e., belonging to the backbone of the MGA alignment, but found in variable segments of the MAUVE alignment) was counted. This was 31.4 kb for seed 15 and 30.2 kb for seed 19. These results showed that a seed size of 19 afforded slightly better performance and we therefore decided to use this seed size for MAUVE alignments in the MOSAIC database.

**Table 1:** MAUVE parameter setup using the collinear genomes of *E. coli *MG1655 and Sakai strains.

**a**								
	**MGA**	**MAUVE**						

Min_rec_gap_length		200 (default)	1000	5000	10000			

Number of backbone segments with a length ≤30 bp	37	588	242	93	45			

Total number of backbone segments	617	1363	959	782	717			

**b**								

	**MGA**	**MAUVE**						

Weight		57 (default)	500	1000	2000	3000	5000	10000

Number of LCB	1	113	47	25	10	4	4	1

Table 1a – Effect of the minimal_recursive_gap_length (Min_rec_gap_length) on backbone fragmentation (seed = 19 and weight=default). Table 1b – Effect of weight on the number of LCBs (min_rec_gap_len = 5000 and seed = 19).

#### LCB analysis

The number and sizes of LCBs produced in MAUVE pairwise alignments were analyzed to detect the presence of rearrangements in one of the aligned genomes (Table S1, in Additional File 1.xls). The idea was to re-align genomes that had not undergone major rearrangements using the MGA aligner, because MGA was used in previous releases of MOSAIC and is more accurate than are alternatives for collinear genomes (our unpublished observations). Genomes were considered as collinear if, first, only one LCB was produced and was not inverted or, second, several LCBs were produced but none of the inverted or translocated LCBs exceeded a threshold of 20 kb in length. This limit of 20 kb was empirically chosen to avoid the detection of too many short rearrangements that are not necessarily significant. For example, the 6.7 kb "translocation" detected by MAUVE in the pairwise *E. coli *comparison described above is considered to be better classified as a "variable segment" than as a translocated backbone segment, as bacteriophages contribute significantly to horizontal transfer. It should be noted that this choice results in a comparison strategy in which only large rearrangements are taken into account; short regions undergoing rearrangements will therefore be classified as variable segments in MOSAIC. LCB analysis of the 704 pairwise alignments obtained from GR release 96 (as described in Table S1) led us to consider 257 genome pairs as collinear and to realign them using MGA.

Maximal multiple genome alignments (i.e., the multiple alignment corresponding to the alignment of all available strains) were then performed using either MGA or MAUVE for species including more than two sequenced strains. For each species, if any of the aligned pairs of genomes was not collinear, the maximal multiple alignment was performed using MAUVE; otherwise the alignment was achieved with MGA. Multiple genome comparisons for 34 species obtained using this strategy are listed in Table 2.

**Table 2:** The 35 maximal multiple chromosome alignments included in the current release of MOSAIC.

**Species**	**#genomes^(1)^**	**Type^(2)^**	**#LCB^(3)^**	**Backbone coverage^(4)^**
*Acinetobacter baumannii*	*4*	*MAUVE*	*33*	*64,73%*

*Actinobacillus pleuropneumoniae*	*3*	*MAUVE*	*5*	*90,65%*

*Bacillus anthracis*	*3*	*MGA*	*-*	*88,61%*

*Bacillus cereus*	*3*	*MGA*	*-*	*71,58%*

*Burkholderia cenocepacia K1*	*3*	*MAUVE*	*4*	*76,92%*

*Burkholderia cenocepacia K2*	*3*	*MAUVE*	*6*	*85,66%*

*Campylobacter jejuni*	*5*	*MAUVE*	*9*	*78,36%*

*Chlamydia pneumoniae*	*4*	*MAUVE*	*2*	*99,64%*

*Chlamydia trachomatis*	*4*	*MAUVE*	*2*	*98,57%*

*Clostridium perfringens*	*3*	*MGA*	*-*	*77,52%*

*Corynebacterium glutamicum*	*3*	*MAUVE*	*4*	*85,11%*

*Coxiella burnetii*	*3*	*MAUVE*	*20*	*94,58%*

*Ehrlichia ruminantium*	*3*	*MGA*	*-*	*94,68%*

*Escherichia coli*	*13*	*MAUVE*	*12*	*68,35%*

*Francisella tularensis*	*6*	*MAUVE*	*55*	*83,49%*

*Haemophilus influenzae*	*4*	*MAUVE*	*19*	*83,46%*

*Helicobacter pylori*	*4*	*MAUVE*	*16*	*80,88%*

*Lactococcus lactis*	*3*	*MAUVE*	*5*	*61,59%*

*Legionella pneumophila*	*4*	*MAUVE*	*8*	*80,37%*

*Methanococcus maripaludis*	*4*	*MAUVE*	*6*	*69,79%*

*Mycobacterium tuberculosis*	*3*	*MAUVE*	*4*	*99,02%*

*Mycoplasma hyopneumoniae*	*3*	*MAUVE*	*3*	*91,11%*

*Neisseria meningitides*	*4*	*MAUVE*	*14*	*78,81%*

*Pseudomonas aeruginosa*	*3*	*MAUVE*	*6*	*80,61%*

*Pseudomonas syringae*	*3*	*MAUVE*	*31*	*61,39%*

*Shewanella baltica*	*3*	*MAUVE*	*14*	*79,29%*

*Staphylococcus aureus*	*14*	*MAUVE*	*1*	*83,50%*

*Streptococcus agalactiae*	*3*	*MGA*	*-*	*84,65%*

*Streptococcus pneumoniae*	*5*	*MAUVE*	*3*	*82,31%*

*Streptococcus pyogenes*	*12*	*MAUVE*	*5*	*80,81%*

*Xanthomonas campestris*	*4*	*MAUVE*	*11*	*54,60%*

*Xanthomonas oryzae*	*3*	*MAUVE*	*21*	*85,82%*

*Xylella fastidiosa*	*4*	*MAUVE*	*9*	*84,26%*

*Yersinia pestis*	*3*	*MAUVE*	*50*	*96,37%*

*Yersinia pseudotuberculosis*	*4*	*MAUVE*	*8*	*89,85%*

(1) Number of aligned genomes.

(2) Type of aligner (MGA or MAUVE).

(3) Number of Locally Collinear Blocks for MAUVE alignments.

(4) Mean ratio of backbone length to genome length.

#### Post-processing of alignments

In a fourth step, MGA and MAUVE pairwise and maximal alignments were post-processed to perform genome segmentation in backbone and variable segments, and database integration. The new term "*variable segments*" was chosen in preference to the previous "*loop" *descriptor, to avoid any ambiguity with respect to secondary structure. For MGA alignments, segmentation was performed as described previously [9]. For MAUVE alignments, backbone and variable segments were defined in a similar manner, except that segmentation was performed for each LCB. Briefly, regions not belonging to an "anchor" (i.e., inexact ungapped seeds), as defined by MAUVE, and less than 10 kb long, were aligned using ClustalW [12], and alignments were automatically inspected. A region was considered to be backbone if all pairwise comparisons yielded more than 76% identity, with never more than 20 consecutive gaps. In all other cases, the entire region was considered to represent a variable segment. MAUVE also generates regions unique to one genome; these are termed "insertions". These were always considered to be variable segments. Note that the term "insertion", chosen by MAUVE, does not necessarily imply that the region in question was acquired by an insertion event.

Finally, several indices were computed for each genome segmentation, including the backbone coverage, the numbers and sizes of backbones and variable segments, and the numbers and sizes of LCBs. Comparisons resulting in low backbone coverage (i.e., lower than 50%) were excluded from the database at this point (see grey rows in Table S1). This affected the pairwise genome alignments of 22 bacterial species including *Buchnera aphidicola*, *Chlorobium phaeobacteroides, Orientia tsutsugamushi, Ralstonia eutropha*, and *Wolbachia pipientis *(for these five species all pairwise alignments are excluded and consequently these species are not present in the current release of the database) and some of the pairwise alignments of the six species of the *Burkholderia *genus, *Campylobacter jejuni, Clostridium botulinum, Leptospira biflexa *and *L. borgpetersenii, Prochlorococcus marinus, Pseudomonas putida, Rhodobacter sphaeroides, Rhodopseudomonas palustris, Synechococcus elongatus, Vibrio cholerae*, and *Yersinia pestis*. These species or groups of species include more divergent genomes that will need to be compared, in future, with a dedicated method.

### Database design and genome alignments

The MOSAIC database is implemented on the relational management database system PostgreSQL (version 8.2.4). The Web interface is designed using the standard Perl modules DBI and CGI.

Genome alignments were processed on a cluster of 160 CPU either with MGA version 2003-03-18 or with MAUVE version 1.2.3. MGA parameters were set up as follows: l = 50–20 and gl = 3000. MAUVE parameter settings were: Seed-size = 19, island-size = 20, backbone-size = 20, max-backbone-gap = 20, gapped-aligner=clustal, max-gapped-aligner-length = 10000, min-recursive-gap-length = 5000, and weight = 5000.

### Improvements to the Web interface

Compared to the previous version, the updated MOSAIC database provides several improvements in the Web interface. First, the "Genome Comparison Viewer" (performed using MuGeN [13]) now shows a global view of rearrangements through the visualization of the LCB structure of all compared genomes. Once an LCB is chosen on the first genome, it is easy to browse the Backbone/Variable Segment structure inside the selected LCB in all compared genomes. When the compared genomes do not show rearrangements, the unique LCB is displayed (as a single purple block) and needs to be selected to access the Backbone/Variable segment structure. Second, the "Circular Map Viewer" (developed using CGView [14]), now allows the user to obtain an interactive circular visualization of the Backbone/Variable Segment structure of a particular chromosome. Third, Specific facilities are provided to visualize and extract coordinates and sequences of "Intervals" (defined by MAUVE as LCBs, and including "Insertions"; see above for insertion definition) in any set of genomes compared with MAUVE.

## Utility and discussion

### Access to comparisons through the Web interface

**Figure 2:**
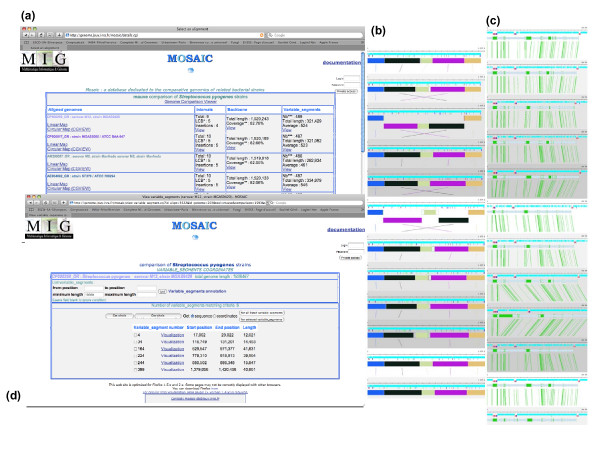
**Example of access to a genome comparison through the MOSAIC Web interface**. Twelve *Streptococcus pyogenes *strains are compared. (a) Main MOSAIC Table describing the general properties of the comparison. A click on the "genome comparison viewer" link gives access to the graphical overview of the five LCBs shown in (b). Selection by clicking on any LCB of the first genome allows the user to zoom in to visualize the backbone/variable segment organization resulting from the alignments, as shown in (c). Backbone regions are shown as grey bars, and variable segments as green bars; genome annotations are superimposed (genes in blue, tRNAs in red). From the main Table (a), access to browse Backbones, Intervals, or Variable Segments [as shown in (d)], is provided.

The main access for browsing MOSAIC bacterial genome comparisons directs the user to choose a species in the MOSAIC main page. Figure 2 presents examples of genome comparison visualization of 12 *Streptococcus pyogenes *genomes through the species access mode.

Once a species is selected, the list of MOSAIC pairwise and multiple genome comparisons available for this species is displayed. The user can then select a comparison to obtain a Table describing the general properties of the comparison (Figure 2a). This table provides the length and backbone coverage, as well as the number, cumulative length, and average length of variable segments, for all aligned genomes. If the comparison was obtained using MAUVE, an additional column lists the number of "Intervals" detected by the alignment procedure. Intervals include LCBs and "Insertions".

The table also gives access to graphical visualization of the aligned genomes. Figure 2b shows an example of the graphical global representation of all aligned genomes using the Genome Comparison Viewer. Once the global view of all aligned chromosomes is generated with the viewer, it is possible to click on each LCB of the first chromosome and then to browse collinear regions of all compared chromosomes. This allows visualization of the backbone and variable segments, together with genome annotations as shown in Figure 2c. Lastly, the table includes links to the detailed list of backbones, variable segments, and, when available, intervals, via the item "View". By following the links it is possible to download these elements in various formats (Figure 2d).

### Case study

Using MOSAIC to compare 12 *S. pyogenes *chromosomes, one can observe that the chromosomes are mostly collinear, with the notable exception of large inversions in strains Manfredo and SSI-1 (Figure 2b). This comparison allowed us to define a backbone 1,500 kb long corresponding to approximately 80% of the total length of the compared chromosomes. The backbone is interrupted by about 480 variable segments whose lengths vary from 20 bp (the MOSAIC minimal threshold) to about 40 kb. In strain MGAS9429, the largest variable segment is 41 kb in length. Using the "visualisation" command (Figure 2d), and the annotation data provided when clicking on each Open Reading Frame (ORF), one can observe that this segment contains numerous ORFs annotated as bacteriophage proteins, indicating that the region may represent integration of a prophage.

## Conclusion

The MOSAIC database aims to provide a powerful resource permitting systematic chromosome comparisons of related bacterial strains.

MOSAIC currently includes chromosome comparisons of 78 bacterial species. MOSAIC has been used to perform 493 pairwise chromosome comparisons (147 processed with MGA and 346 processed with MAUVE), and 35 multiple maximal chromosome comparisons (5 processed with MGA and 30 processed with MAUVE). Of particular interest, three species include multiple alignments of many strains. These are *Staphylococcus aureus *(14 genomes compared), *E. coli/Shigella *(13 genomes compared), and *S. pyogenes *(12 genomes compared). Except for a few cases for which genomes are too divergent to be aligned (such as in strains of the endosymbiotic species *Buchnera aphidicola*), all bacterial species for which at least two strains are sequenced are included in MOSAIC. The MOSAIC database can be used for a variety of comparative analyses and applications. To date, the database has been employed to predict motifs involved in bacterial chromosome maintenance in four species by analyzing backbone regions [15]. MOSAIC has also being used to analyze the mechanisms of genetic variability in *E. coli*, *S. aureus*, and *S. pyogenes*, and to analyze recombination, using an alignment of backbones obtained from a comparison of 20 *E. coli/Shigella *strains sequenced by the ColiScope consortium [16].

Future developments will include complete automation of releases, comparison of divergent genomes using a dedicated strategy, integration of statistical criteria for evaluation of chromosome comparisons, and development of Web services to provide standard exchanges with other resources.

## Availability and requirements

The database is available at .

This web site is optimized for Firefox 1.5.x and 2.x. Note that some pages may not be correctly displayed with other browsers.

## Authors' contributions

HC and MEK conceived the study. HC and AG designed the database and developed the Perl scripts. CC developed the Web interface. MAP and MEK performed the set up of MAUVE parameters and contributed to writing the manuscript. JB computed the alignments and developed the rearrangement test. HC drafted the manuscript. All authors read and approved the final manuscript.

## Supplementary Material

Additional File 1 **Table S1- Pairwise genome alignments performed using bacterial genomes downloaded from Genome Reviews**. The Table lists the 704 pairwise chromosome alignments performed with MAUVE at the intra-species level. Columns list the alignment identifier (Id), the species, the accession number (Access), the strain name (Strain) for the two aligned chromosomes, the total number of Locally Collinear Blocks (Total LCBs) produced by MAUVE, the number of Inverted LCBs (Inv. LCBs), the maximal size of the inverted LCBs (Max size Inv. LCBs), the number of translocated LCBs which are more than 20 kb in length (TR>20 kb), alignments flagged to be realigned with MGA, and the mean percentages of the chromosomes assigned to backbones after integration in the MOSAIC database (Backbone coverage). Comparisons with low backbone coverage (i.e., lower than 50%) are shown as grey rows. ^1^*Shigella *chromosomes were compared with *E. coli *chromosomes because these strains can be considered as belonging to the same species [17]. Click here for file
